# Wide-field swept-source OCT angiography of the periarterial capillary-free zone before and after anti-VEGF therapy for branch retinal vein occlusion

**DOI:** 10.1186/s40662-022-00297-z

**Published:** 2022-07-02

**Authors:** Wenyi Tang, Wei Liu, Jingli Guo, Lili Zhang, Gezhi Xu, Keyan Wang, Qing Chang

**Affiliations:** 1 Department of Ophthalmology, Eye and ENT Hospital of Fudan University, 83 Fenyang Road, Shanghai, 200031 China; 2 Shanghai Key Laboratory of Visual Impairment and Restoration, Fudan University, Shanghai, China; 3 NHC Key Laboratory of Myopia (Fudan University), Laboratory of Myopia, Chinese Academy of Medical Sciences, Shanghai, China

**Keywords:** Swept-source optical coherence tomography angiography, Branch retinal vein occlusion, Periarterial capillary free zone, Anti-VEGF treatment, Prognosis

## Abstract

**Background:**

The aim of the study was to investigate the changes in the periarterial capillary-free zone (paCFZ) after anti-vascular endothelial growth factor (VEGF) therapy in patients with branch retinal vein occlusion (BRVO) by wide-field swept-source optical coherence tomography angiography (SS-OCTA) and assess their associations with clinical outcomes.

**Methods:**

In this retrospective observational study of 54 treatment-naïve BRVO patients with macular edema, we reviewed the findings of 12 × 12 mm^2^ SS-OCTA at baseline, 3, 6, and 12 months after intravitreal ranibizumab injections. The paCFZ and major retinal artery areas were measured on SS-OCTA images. The paCFZ area to artery area (P/A) ratio was calculated.

**Results:**

The paCFZ areas and P/A ratios of first- and second-order arteries were significantly greater in BRVO eyes than in contralateral eyes (all *P* < 0.01), but there were no differences in the first- and second-order artery areas (*P* = 0.20 and 0.25, respectively). The paCFZ areas and P/A ratios decreased significantly at 3, 6, and 12 months after anti-VEGF therapy (all *P* < 0.01). The baseline P/A ratio was significantly correlated with the baseline best-corrected visual acuity (BCVA), central retinal thickness, and their improvements at 3, 6, and 12 months (all *P* < 0.05). Baseline BCVA and P/A ratios of first- and second-order arteries were independently associated with the final BCVA in multivariate linear regression.

**Conclusions:**

Wide-field SS-OCTA shows that anti-VEGF therapy can lead to a significant improvement in the paCFZ parameters in BRVO. Smaller baseline P/A ratios on SS-OCTA tend to predict better visual outcomes at 12 months after anti-VEGF therapy.

**Supplementary Information:**

The online version contains supplementary material available at 10.1186/s40662-022-00297-z.

## Background

Branch retinal vein occlusion (BRVO) is a common retinal vascular disease worldwide, where macular edema is the main cause of visual impairment [1]. Macular edema is characterized by central retinal thickening caused by vascular leakage and fluid accumulation, which is triggered by the release of mediators, particularly vascular endothelial growth factor (VEGF) [2]. Large randomized clinical trials have demonstrated that intravitreal injection of anti-VEGF agents can reduce macular edema and improve visual acuity. Thus, anti-VEGF agents have become the standard therapy for patients with macular edema secondary to BRVO [3, 4]. However, some patients show resistance to anti-VEGF therapy and have poor prognosis [5]. To date, few clinical biomarkers that have been identified reflect disease activity and can be used to predict prognosis [6].

Fundus fluorescein angiography (FA) is considered the gold-standard method for evaluating retinal microvascular abnormalities and macular nonperfusion. Optical coherence tomography angiography (OCTA), a recently developed technique, offers many advantages for non-invasive retinal evaluation including high resolution and layered imaging of the posterior pole vasculature [7]. An increasing number of studies have utilized OCTA to evaluate the microvascular dysfunction in BRVO eyes with qualitative and quantitative analyses [8, 9]. The retinal periarterial capillary-free zone (paCFZ), first described by His in 1880, is a physiologically avascular area surrounding the retinal arteries exclusively in the superficial capillary plexus layer [10, 11]. Its formation is based on oxygen saturation during embryonic development because the retinal arteries contain a high concentration of oxygen, and vasoobliteration of capillaries in the periarterial area can be induced upon exposure to hyperoxia [12]. We previously reported that the retinal paCFZ was enlarged along unaffected major retinal arteries in eyes with BRVO [13]. Li et al. reported that the paCFZ was significantly larger in patients with severe nonproliferative diabetic retinopathy (DR) compared with those in normal subjects [14]. Based on those studies, it was suggested that the paCFZ might be a useful biomarker for monitoring retinal diseases associated with changes in the retinal microvasculature [15]. However, those studies were cross-sectional in design and, to our knowledge, no studies have reported the longitudinal changes in the paCFZ in retinal vascular disorders.

In this retrospective study, we assessed the changes in the paCFZ before and after anti-VEGF therapy in patients with macular edema due to BRVO by using wide-field swept-source OCTA (SS-OCTA) images. We also examined the associations between these changes and other clinical parameters, including macular thickness and visual acuity, to investigate whether paCFZ parameters can predict the response and functional outcomes after anti-VEGF treatment in BRVO eyes.

## Methods

### Patients

This retrospective, observational study was approved by the institutional review board of the Eye and ENT Hospital of Fudan University (Shanghai, China), and it followed the tenets of the Declaration of Helsinki. The study included consecutive patients with macular edema due to BRVO who were treated with intravitreal ranibizumab (IVR; 0.5 mg, Lucentis®; Novartis Pharma AG, Basel, Switzerland) at the Eye and ENT Hospital of Fudan University between December 2018 and September 2020. Healthy volunteers with no retinal disorders were also recruited. Written informed consent for the treatment and the use of clinical data for research aims was obtained from all the participants.

The inclusion criteria included the following: (1) unilateral treatment-naïve macular edema secondary to BRVO diagnosed based on fundus examinations and FA; (2) symptom duration of less than 3 months; (3) minimum follow-up of 12 months; and (4) SS-OCTA images obtained before and after IVR. The exclusion criteria were as follows: (1) presence of other coexisting retinal disorders such as DR; (2) severe media opacities that hindered fundus examination; (3) high myopia [− 6 diopters (D) or lower]; (4) high hyperopia (+ 5.0 D or greater); (5) history of intravitreal injection of corticosteroids or anti-VEGF drugs; (6) history of laser photocoagulation or vitreoretinal surgery; (7) presence of any retinal disease or media opacity in the contralateral eye; and (8) poor-quality SS-OCTA images (signal strength less than 7) or obvious motion artifacts in either eye. The control group consisted of subjects with unnoticeable findings on fundus examinations and the study eye was randomly selected.

### Ocular examinations and treatment

The patients’ demographics and medical history were collected from their medical records. All patients underwent complete ophthalmic examinations, which included the measurement of best-corrected visual acuity (BCVA) and intraocular pressure (IOP), manifest refraction (NIDEK RT-5100; NIDEK, Tokyo, Japan), slit-lamp biomicroscopy, fundus photography, spectral-domain optical coherence tomography (SD-OCT; Spectralis HRA + OCT; Heidelberg Engineering, Heidelberg, Germany), SS-OCTA (Plex Elite 9000; Carl Zeiss Meditec, Inc., Dublin, CA, USA), and ultra-wide-field FA (Optos 200Tx imaging system; Optos PLC, Dunfermline, UK). Ischemic BRVO was defined as BRVO with a capillary nonperfusion area of greater than 5 disk diameters on FA and nonischemic BRVO was defined as BRVO with a capillary nonperfusion area of ≤ 5 disk diameters [16]. The occlusion area in BRVO eyes defined as the quadrant affected by occluded venules was recorded after fundus examination. BCVA was measured using the Early Treatment Diabetic Retinopathy Study visual acuity testing charts. Central retinal thickness (CRT) was measured in a circular region of 1 mm diameter centered on the fovea using the built-in software. Macular edema was defined as a CRT of ≥ 300 μm or intraretinal or subretinal fluids in the parafoveal region on SD-OCT [17]. The patients received three injections at intervals of 1 month and subsequent pro re nata (PRN) injections at monthly visits, alongside comprehensive ophthalmic examinations, which included BCVA, slit-lamp biomicroscopy, fundus photography, SD-OCT, and SS-OCTA. Additional IVR injections were administered if intraretinal or subretinal fluid was observed, or if the CRT had increased by more than 50 μm from the lowest recorded value.

### SS-OCTA analysis

SS-OCTA scans (3 × 3 mm^2^ and 12 × 12 mm^2^) centered on the fovea were performed with a central wavelength of 1050 nm and an A-scan speed of 100 kHz. The axial and transverse resolutions were 6.3 μm and 20 μm, respectively. Each 3 × 3 mm^2^ or 12 × 12 mm^2^ volume scan comprised 500 A-scans per B-scan with a total of 500 B-scan locations**.** All OCTA B-scans were examined and manual adjustments were performed for segmentation errors if present.

The area of the foveal avascular zone (FAZ) of the superficial capillary plexus layer on the 3 × 3 mm^2^ SS-OCTA images were measured with the software ImageJ (http://imagej.nih.gov/ij/) as described previously [13, 18]. The paCFZ parameters in BRVO eyes were measured in the horizontally opposed quadrant, as any hemorrhage or edema in the involved quadrant was likely to make the measurement difficult and unreliable. The corresponding quadrants of the contralateral eyes and the healthy control eyes were also measured. We used custom software (Medraw; Image Medraw Technology Co., Ltd., Shanghai, China) to semiautomatically identify the paCFZ areas and the corresponding artery areas of the major retinal arteries on 12 × 12 mm^2^ en face images of the superficial capillary plexus layer. We previously reported that the software and measurement approach had high reproducibility and repeatability in healthy and BRVO eyes [13]. Briefly, two trained graders (WYT and JLG) identified the first- and second-order arteries on the fundus photographs and SS-OCTA images, as previously reported [19]. Next, the SS-OCTA images were imported into the software and a seed point was added to the paCFZ or the corresponding artery by the graders independently. The software detected the pixel of the seed point (SeedPix). The target area was defined as the area which contained pixels between 0 and SeedPix surrounding the seed point, and the software calculated the pixels of the target area (AreaPix) automatically. The value was then converted to square millimeters using the formula Area (mm^2^) = AreaPix × [(realH × realW)/(pixH × pixW)], where the real height (realH), real width (realW), pixel height (pixH), and pixel width (pixW) were 12 mm, 12 mm, 1024 pixels, and 1024 pixels, respectively, in the 12 × 12 mm^2^ SS-OCTA images. To reduce the potential influence of the individual variability in the branches of the major retinal arteries, the paCFZ area to artery area (P/A) ratio was calculated. The values of the paCFZ and the corresponding artery areas obtained by two graders (WYT and JLG) were averaged for analysis.

### Statistical analyses

Data were presented as the number or mean ± SD. Paired t-tests and unpaired t-tests were used to compare data between the BRVO eyes and the contralateral eyes and between the contralateral eyes and the healthy control eyes, respectively. Intraclass correlation coefficients (ICCs) were used to test intra-observer repeatability and inter-observer reproducibility. CRT, BCVA, artery areas, and paCFZ parameters (paCFZ area and P/A ratio) at baseline and at 3, 6, and 12 months after anti-VEGF treatment were compared using repeated-measures One-way analysis of variance (ANOVA) with Geisser-Greenhouse correction. The Bonferroni post hoc test was then used to compare the post-treatment values with the baseline values in cases presenting with significant fluctuations. Associations between baseline CRT and BCVA, improvements in CRT and BCVA, and the number of IVR injections with the P/A ratio were evaluated using Pearson’s correlation coefficients. Uni- and multi-variate linear regression analyses were used to determine which baseline variables were significantly associated with the BCVA at 12 months. Variables of *P* < 0.05 in the univariate analysis were included in the multivariate analysis by a stepwise method. SPSS Statistics (version 21; SPSS, Chicago, IL, USA) was used to perform the statistical analyses. Significance was set at a *P* value of less than 0.05.

## Results

Fifty-four patients and 30 control subjects were included in this study; their clinical characteristics are summarized in Table 1. No significant difference was observed between the BRVO and the control groups pertaining to the demographics and systemic disease at baseline. Thirty-six patients had ischemic BRVO, and 18 patients had nonischemic BRVO. The IOP (*P* = 0.15) and refractive error (*P* = 0.10) were not significantly different between the BRVO eyes and the contralateral eyes. The BRVO eyes had worse BCVA (*P* < 0.01), thicker CRT (*P* < 0.01) and larger FAZ areas (*P* < 0.01) than the contralateral eyes. The paCFZ areas and the P/A ratios of the first- and second-order arteries were significantly greater in BRVO eyes than in the contralateral eyes (all *P* < 0.01). The first- and second-order artery areas (*P* = 0.20 and 0.25, respectively) were not significantly different between the BRVO eyes and the contralateral eyes. Furthermore, there were no significant differences in BCVA, CRT, FAZ area, paCFZ area, artery area and the ratio of the paCFZ area to the artery area between the contralateral eyes and the healthy control eyes (all *P* > 0.05, Table 1). The intra-observer agreement and inter-observer agreement were extremely high for the baseline paCFZ and areas of the first- and second-order arteries, with ICCs of more than 0.9 (Additional file 1: Table S1). The mean number of IVR injections administered over 12 months was 4.98 ± 2.17 (range: 3–10).

**Table 1:** Baseline clinical data of patients with BRVO and healthy control participants in this study

Parameters	BRVO eyes (n = 54)	Fellow eyes (n = 54)	Healthy control eyes (n = 30)	*P* (BRVO vs. fellow)	*P* (fellow vs. control)
Age (years, range)	60.56 ± 9.18 (45–79)		60.92 ± 5.22 (50–73)	NA	0.85
Sex (male/female)	35/19		18/12	NA	0.66
Eye dominance (OD/OS)	35/19	19/35	14/16	NA	0.30
Hypertension (+/−)	31/23		13/17	NA	0.22
Mean systolic pressure (mmHg)	132.81 ± 13.67		129.68 ± 10.95	NA	0.32
Mean diastolic pressure (mmHg)	78.31 ± 8.78		79.52 ± 5.65	NA	0.53
IOP (mmHg)	14.41 ± 3.52	15.06 ± 3.00	15.84 ± 2.75	0.15	0.28
Refractive error (SE, diopter)	− 0.15 ± 1.52	− 0.42 ± 1.55	− 0.24 ± 1.28	0.10	0.63
Duration of symptoms (months)	1.50 ± 1.03	NA	NA	NA	NA
Type (ischemia/non-ischemia)	36/18	NA	NA	NA	NA
Occlusion area (ST/IT)	38/16	NA	NA	NA	NA
Quadrants measured (ST/IT)	16/38	16/38	9/21	NA	0.97
BCVA (ETDRS letters, Snellen equivalent)	53.56 ± 16.09 (20/90)	84.30 ± 2.49 (20/21)	84.60 ± 1.04 (20/20)	< 0.01	0.56
CRT (μm)	581.70 ± 191.90	257.41 ± 26.21	260.60 ± 23.17	< 0.01	0.60
FAZ area (mm^2^)	0.41 ± 0.15	0.35 ± 0.09	0.32 ± 0.10	< 0.01	0.16
First-order artery					
PaCFZ area (mm^2^)	0.54 ± 0.18	0.45 ± 0.15	0.42 ± 0.16	< 0.01	0.53
Artery area (mm^2^)	0.49 ± 0.20	0.53 ± 0.14	0.51 ± 0.16	0.20	0.50
P/A ratio	1.14 ± 0.22	0.84 ± 0.22	0.83 ± 0.16	< 0.01	0.78
Second-order artery					
PaCFZ area (mm^2^)	0.96 ± 0.48	0.75 ± 0.25	0.73 ± 0.25	< 0.01	0.78
Artery area (mm^2^)	0.60 ± 0.38	0.65 ± 0.23	0.64 ± 0.24	0.25	0.88
P/A ratio	1.83 ± 0.55	1.25 ± 0.53	1.18 ± 0.29	< 0.01	0.59
No. of IVR injections (range)	4.98 ± 2.17 (3–10)	NA	NA	NA	NA

*BRVO* = branch retinal vein occlusion; *NA* = not applicable; *IOP* = intraocular pressure; *SE* = spherical equivalent; *ST* = superotemporal; *IT* = inferotemporal; *BCVA* = best-corrected visual acuity; *ETDRS* = Early Treatment Diabetic Retinopathy Study; *CRT* = central retinal thickness; *FAZ* = foveal avascular zone; *paCFZ* = periarterial capillary-free zone; *P/A* = paCFZ area to artery area; *No.* = number; *IVR* = intravitreal ranibizumab

**Fig. 1:**
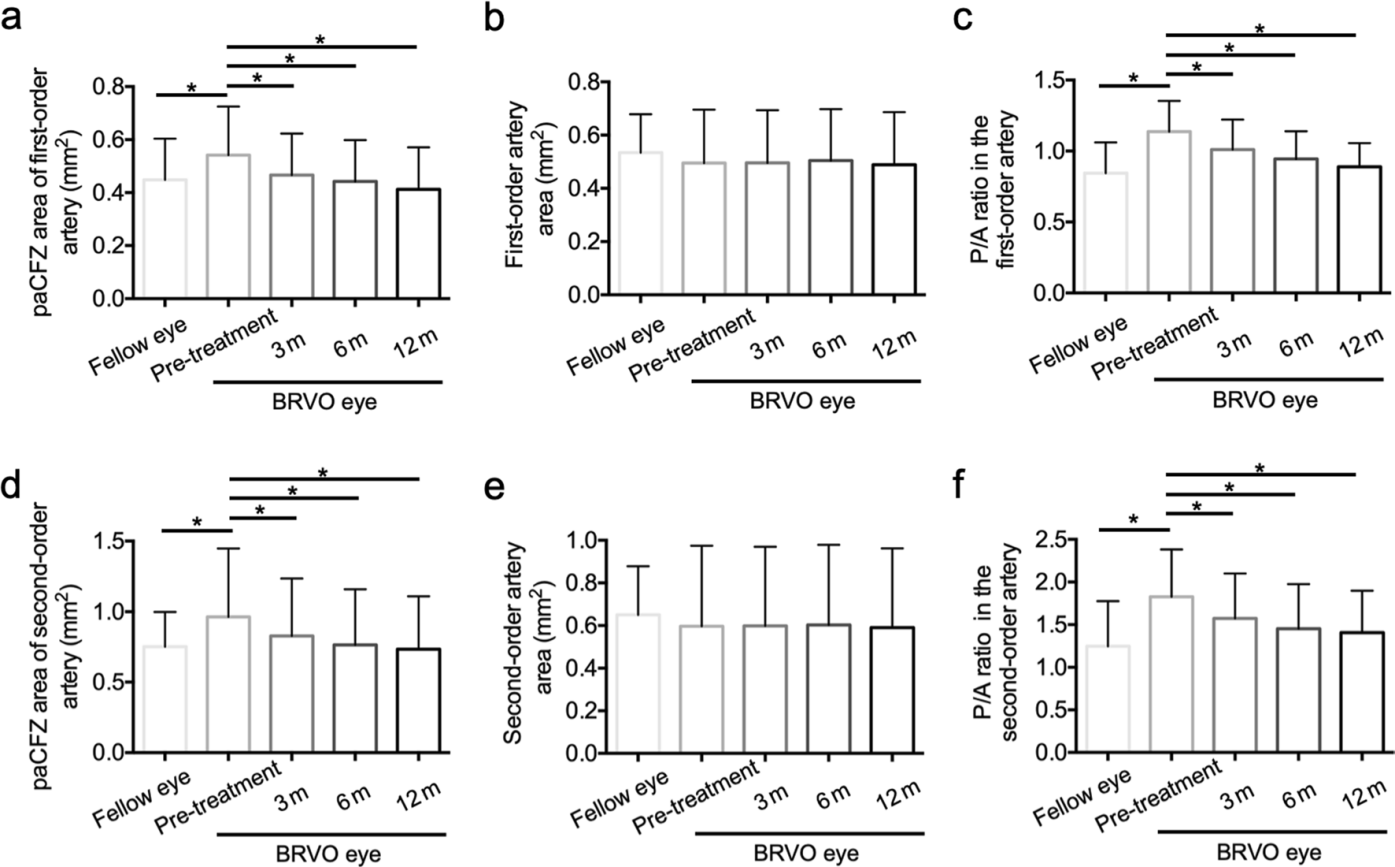
The periarterial capillary-free zone (paCFZ) areas, artery areas and paCFZ area to artery area (P/A) ratio of the first-order (**a**–**c**) and second-order (**d**–**f**) arteries in the unaffected quadrants of branch retinal vein occlusion (BRVO) eyes at baseline and 3, 6 and 12 months after intravitreal ranibizumab (IVR) injections. Before treatment, the BRVO eyes demonstrated significantly larger paCFZ areas (*P* < 0.01, *P* < 0.01), paCFZ/artery (P/A) ratios (*P* < 0.01, *P* < 0.01) of the first-order (**a**, **c**) and second-order (**d**, **f**) arteries compared with the fellow eyes, while the corresponding artery areas (**b**, **e**) did not show significant difference, respectively (*P* = 0.20, *P* = 0.25). After IVR injections, the paCFZ areas (*P* < 0.01, *P* < 0.01) and P/A ratios (*P* < 0.01, *P* < 0.01) demonstrated remarkable decreases in the first-order (**a**, **c**) and second-order (**d**, **f**) arteries. Pairwise comparisons between baseline and 3, 6, and 12 months after treatment revealed significant reductions in the paCFZ areas (all *P* < 0.01) and P/A ratios (all *P* < 0.01) of the first-order (**a**, **c**) and the second-order (**d**, **f**) arteries. The paCFZ areas and P/A ratios returned almost to the levels of the healthy fellow eyes (**a**, **c**, **d**, **f**). However, the artery areas were not significantly decreased in the first-order artery (**b**, *P* = 0.05) or the second-order artery (**e**, *P* = 0.20), respectively. *P < 0.05

**Fig. 2:**
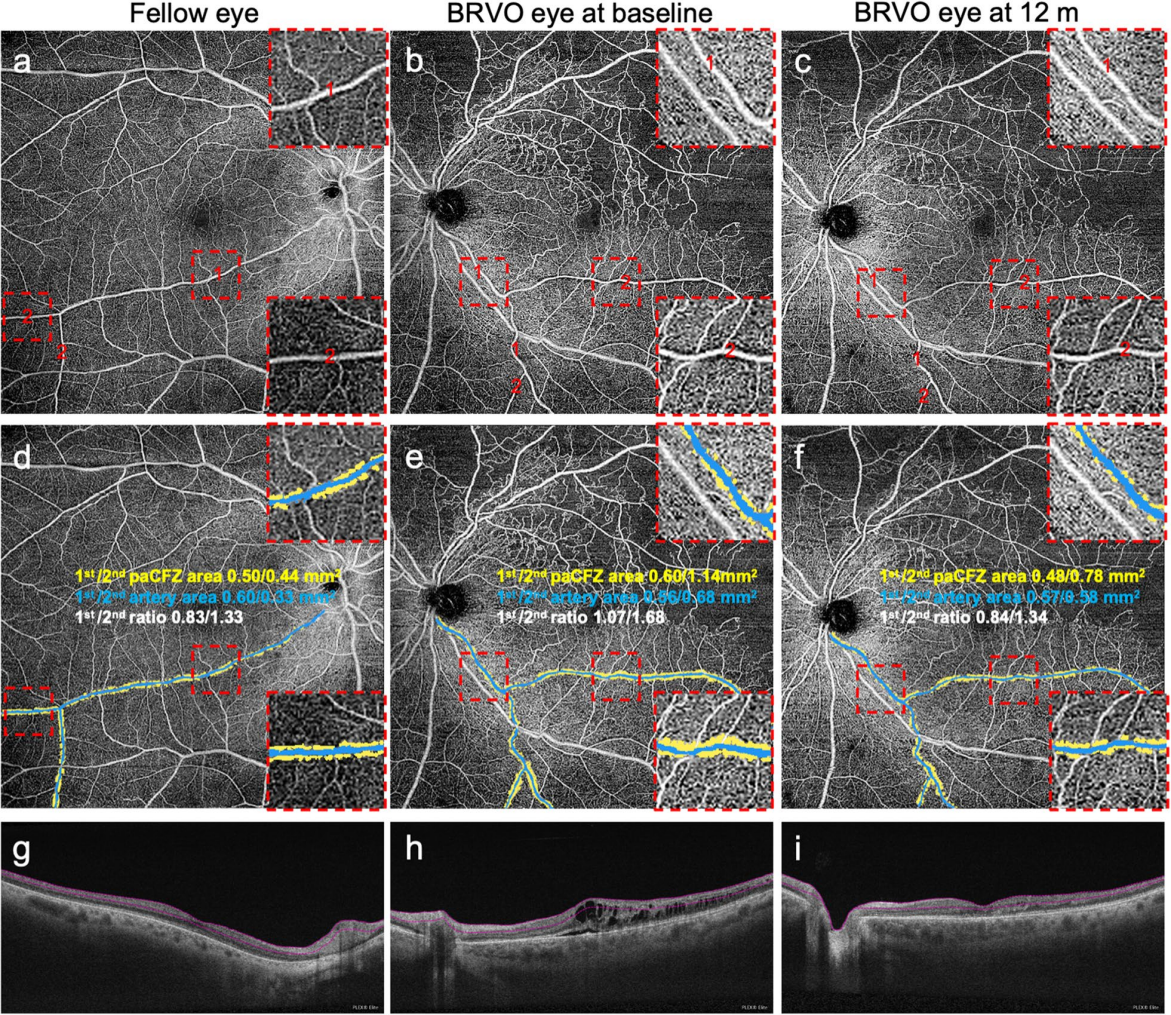
Representative swept-source optical coherence tomography angiography (SS-OCTA) images and measurements of the periarterial capillary-free zone (paCFZ) areas and corresponding artery areas, including calculation of the paCFZ/artery (P/A) ratios in a patient with branch retinal vein occlusion (BRVO). The patient’s best-corrected visual acuity of the affected eye was 60 letters at baseline and 75 letters at 12 months after intravitreal ranibizumab therapy. **a**–**c** SS-OCTA images of the healthy contralateral eye (**a**), the BRVO eye at baseline (**b**), and the BRVO eye at 12 months after ranibizumab therapy (**c**). The details of the paCFZ areas along the first- and second-order arteries were shown in the magnified squares. **d–f** Measurements of the paCFZ areas and corresponding artery areas shown in **a** (**d**), **b** (**e**) and **c** (**f**). The magnified squares demonstrated the detailed measurement of paCFZ and artery areas. **g**–**i** The corresponding 12 × 12 mm B-scan images of the superficial capillary plexus. **a** The first- and second-order arteries in the inferotemporal quadrant were determined in the healthy contralateral eye. **d** For measurements of the first- and second-order arteries in the healthy contralateral eye, the paCFZ areas were 0.50 mm^2^ and 0.44 mm^2^, respectively, the artery areas were 0.60 mm^2^ and 0.33 mm^2^, respectively, and the P/A ratios were 0.83 and 1.33, respectively. The details of the paCFZ areas along the first- and second-order arteries were shown in the magnified squares. **b** The BRVO eye exhibited retinal nonperfusion in the affected supertemporal quadrant of the retina. **e** For the first- and second-order arteries in the inferotemporal quadrant, the paCFZ areas were 0.60 mm^2^ and 1.14 mm^2^, respectively, the artery areas were 0.56 mm^2^ and 0.68 mm^2^, respectively, and the P/A ratios were 1.07 and 1.68, respectively. **c**, **f** For measurements of the first- and second-order arteries at 12 months after ranibizumab therapy, the paCFZ areas were 0.48 mm^2^ and 0.78 mm^2^, respectively, the artery areas were 0.57 mm^2^ and 0.58 mm^2^, respectively, and the P/A ratios were 0.84 and 1.34, respectively. The paCFZ areas and P/A ratios were greater in the affected eye than in the healthy contralateral eye at baseline, but these parameters decreased with a reduction in macular edema after five intravitreal injections of ranibizumab

Figure 1 and Table 2 show the time-dependent changes in BCVA, CRT, and paCFZ parameters after anti-VEGF therapy. The BCVA improved significantly after therapy, from 53.56 ± 16.09 letters at baseline to 63.37 ± 16.96 letters at 3 months, 63.98 ± 13.48 letters at 6 months, and 65.67 ± 12.89 letters at 12 months (all *P* < 0.01). The CRT decreased significantly from 581.70 ± 191.90 μm at baseline to 283.70 ± 78.02 μm at 3 months, 286.90 ± 85.19 μm at 6 months, and 262.5 ± 47.10 μm at 12 months (all *P* < 0.01). The paCFZ areas and P/A ratios of the first- and second-order arteries decreased significantly during the 12-month follow-up period (all *P* < 0.01, Fig. 2), the values of which returned almost to the levels of the healthy contralateral eyes. Pairwise comparisons between baseline and 3, 6, and 12 months after treatment revealed significant reductions in the paCFZ areas (all *P* < 0.01) and P/A ratios (all *P* < 0.01) of the first- and second-order arteries. However, the areas of the first- (*P* = 0.05) and second-order arteries (*P* = 0.20) did not decrease significantly. The FAZ areas (0.39 ± 0.15 mm^2^) did not significantly change at 12 months after anti-VEGF treatment compared with those at baseline (0.41 ± 0.15 mm^2^, *P* = 0.61). Furthermore, a subgroup analysis demonstrated that the P/A ratios of the first- and second-order arteries were significantly larger in the eyes with ischemic BRVO than nonischemic BRVO at baseline (Table 3). Although the paCFZ areas and P/A ratios were both significantly reduced at 12 months after anti-VEGF treatment in these two subgroups, the P/A ratios of the first- and second-order arteries were still significantly larger in the eyes with ischemic BRVO than nonischemic BRVO (Table 3). The paCFZ areas, artery areas and P/A ratios in the healthy contralateral eyes did not change significantly at 12 months compared with those at baseline, respectively (Additional file 1: Table S2).

**Table 2:** Changes of clinical parameters at 3, 6 and 12 months after anti-VEGF treatment in BRVO eyes

Parameters	BL	3 m	6 m	12 m	*P*			
					RM one-way ANOVA	Bonferroni post hoc test*		
						BL vs. 3 m	BL vs. 6 m	BL vs. 12 m
BCVA (ETDRS letters)	53.56 ± 16.09	63.37 ± 16.96	63.98 ± 13.48	65.67 ± 12.89	< 0.01	< 0.01	< 0.01	< 0.01
CRT (μm)	581.70 ± 191.90	283.70 ± 78.02	286.90 ± 85.19	262.50 ± 47.10	< 0.01	< 0.01	< 0.01	< 0.01
First-order artery								
PaCFZ area (mm^2^)	0.54 ± 0.18	0.47 ± 0.16	0.44 ± 0.16	0.41 ± 0.16	< 0.01	< 0.01	< 0.01	< 0.01
Artery area (mm^2^)	0.49 ± 0.20	0.50 ± 0.20	0.50 ± 0.19	0.49 ± 0.20	0.05	–	–	–
P/A ratio	1.14 ± 0.22	1.01 ± 0.21	0.94 ± 0.20	0.89 ± 0.17	< 0.01	< 0.01	< 0.01	< 0.01
Second-order artery								
PaCFZ area (mm^2^)	0.96 ± 0.48	0.83 ± 0.41	0.76 ± 0.40	0.73 ± 0.38	< 0.01	< 0.01	< 0.01	< 0.01
Artery area (mm^2^)	0.60 ± 0.38	0.60 ± 0.37	0.60 ± 0.38	0.59 ± 0.37	0.20	–	–	–
P/A ratio	1.83 ± 0.55	1.57 ± 0.53	1.45 ± 0.52	1.41 ± 0.49	< 0.01	< 0.01	< 0.01	< 0.01

*VEGF* = vascular endothelial growth factor; *BRVO* = branch retinal vein occlusion; *RM* = repeated measures; *ANOVA* = analysis of variance; *BL* = baseline; *BCVA* = best-corrected visual acuity; *ETDRS* = Early Treatment Diabetic Retinopathy Study; *CRT* = central retinal thickness; *paCFZ* = periarterial capillary-free zone; *P/A* = paCFZ area to artery area

*Adjusted *P* value

**Table 3:** Comparison of paCFZ parameters in the first- and second-order arteries in ischemic and nonischemic BRVO at baseline and 12 months after anti-VEGF treatment

Parameters	BL		P*	12 m		P*	*P* (BL vs. 12 m)**	
	iBRVO (n = 36)	NiBRVO (n = 18)		iBRVO (n = 36)	NiBRVO (n = 18)		iBRVO (n = 36)	NiBRVO (n = 18)
First-order								
PaCFZ area (mm^2^)	0.55 ± 0.18	0.53 ± 0.20	0.74	0.43 ± 0.18	0.43 ± 0.19	0.99	< 0.01	< 0.01
Artery area (mm^2^)	0.47 ± 0.18	0.52 ± 0.18	0.44	0.47 ± 0.18	0.51 ± 0.18	0.39	0.06	0.28
P/A ratio	1.19 ± 0.21	1.04 ± 0.20	0.02	0.92 ± 0.16	0.81 ± 0.16	0.04	< 0.01	< 0.01
Second-order								
PaCFZ area (mm^2^)	1.01 ± 0.54	0.85 ± 0.34	0.24	0.77 ± 0.42	0.66 ± 0.27	0.30	< 0.01	< 0.01
Artery area (mm^2^)	0.58 ± 0.38	0.63 ± 0.38	0.62	0.56 ± 0.37	0.65 ± 0.38	0.45	0.08	0.43
P/A ratio	1.96 ± 0.52	1.57 ± 0.53	0.01	1.55 ± 0.48	1.20 ± 0.46	0.01	< 0.01	< 0.01

*paCFZ* = periarterial capillary-free zone; *BRVO* = branch retinal vein occlusion; *VEGF* = vascular endothelial growth factor; *BL* = baseline; *iBRVO* = ischemic branch retinal vein occlusion; *NiBRVO* = non-ischemic branch retinal vein occlusion; *P/A* = paCFZ area to artery area

*Unpaired t-test

**Paired t-test

The associations between the baseline P/A ratios and the baseline values or the improvements in CRT and BCVA are summarized in Table 4. The baseline P/A ratios were significantly correlated with baseline CRT and baseline BCVA. Furthermore, the baseline P/A ratio was significantly correlated with the improvement in CRT (calculated as the baseline value minus post-IVR value) at 3, 6, and 12 months. The baseline P/A ratio was also significantly correlated with the improvement in BCVA (post-IVR value minus baseline value) at 3, 6, and 12 months. However, the paCFZ and artery areas were not significantly correlated with the baseline values or the improvements in CRT and BCVA (Additional file 1: Table S3). The baseline P/A ratios were significantly correlated with the number of IVR injections during the 12-month observation period (first-order: *r* = 0.293, *P* = 0.032; second-order: *r* = 0.324, *P* = 0.017).

**Table 4:** Simple regression analysis of baseline P/A ratios with central retinal thickness, best-corrected visual acuity, and their improvement at 3, 6, 12 months of the follow-up

Variables	P/A ratio in the first-order artery		P/A ratio in the second-order artery	
	r	*P*	r	*P*
Baseline CRT	0.366	0.006	0.377	0.005
CRT improvement				
At 3 months	0.302	0.027	0.310	0.023
At 6 months	0.312	0.021	0.307	0.024
At 12 months	0.366	0.007	0.363	0.006
Baseline BCVA	− 0.370	0.005	− 0.380	0.004
BCVA improvement				
At 3 months	− 0.677	< 0.001	− 0.682	< 0.001
At 6 months	− 0.304	0.026	− 0.287	0.036
At 12 months	− 0.437	0.001	− 0.445	< 0.001

*P/A* = paCFZ area to artery area; *CRT* = central retinal thickness; *BCVA* = best-corrected visual acuity; *ETDRS* = Early Treatment Diabetic Retinopathy Study

The associations between the baseline clinical features and BCVA at 12 months are shown in Table 5. In univariate regression, better BCVA at 12 months was significantly associated with shorter duration of symptoms (*P* = 0.042), better baseline BCVA (*P* < 0.001), smaller baseline CRT (*P* = 0.026), and smaller baseline P/A ratios of the first- (*P* < 0.001) and second-order arteries (*P* < 0.001). In multivariate linear regression analysis, the baseline BCVA and P/A ratios of the first- and second-order arteries were independently associated with the BCVA at 12 months (*P* < 0.001, 0.003, and 0.041, respectively; Table 5).

**Table 5:** Univariate and multivariate regression analysis for the association between baseline variables and BCVA at 12 months

Variables	Univariate linear regression		Multivariate linear regression	
	r	*P*	β	*P*
Age	− 0.035	0.800	–	–
Sex	− 0.166	0.230	–	–
Hypertension	− 0.099	0.476	–	–
Duration	− 0.278	0.042	− 0.250	0.948
Type (ischemia/non-ischemia)	0.092	0.507	–	–
Baseline BCVA	0.969	< 0.001	0.840	< 0.001
Baseline CRT	− 0.302	0.026	− 0.193	0.876
Baseline FAZ area	− 0.210	0.128	–	–
P/A ratio in the first-order artery	− 0.828	< 0.001	− 0.163	0.003
P/A ratio in the second-order artery	− 0.963	< 0.001	− 0.242	0.041

*P/A* = paCFZ area to artery area; *CRT* = central retinal thickness; *BCVA* = best-corrected visual acuity; *FAZ* = foveal avascular zone

## Discussion

In this study, we demonstrate for the first time that the paCFZ areas and the P/A ratios of the major retinal arteries decreased significantly after anti-VEGF therapy in eyes with macular edema secondary to BRVO. Furthermore, the baseline BCVA and P/A ratio were found to be associated with the long-term visual outcomes after anti-VEGF therapy in these patients.

PaCFZ is closely related to retinal tissue oxygenation, which oxygen and nutrients have to diffuse across to supply the retinal neurons [12, 15]. The pathogenesis of the dynamic changes of the paCFZ in BRVO eyes after anti-VEGF therapy is unclear. One possible explanation is that, after venous occlusion, retinal oxygen consumption decreases and the arterial oxygen saturation increases, which might increase the diffusion distance of oxygen and hence the paCFZ area. Anti-VEGF therapy, which decreases CRT and allows the inner retinal neurons to recover, is associated with increased oxygen and nutrient extraction and decreased oxygen saturation of the arteries, resulting in reductions of the paCFZ areas. Indeed, we observed a remarkable decrease in the CRT and a significant improvement in BCVA following anti-VEGF therapy. Several studies have reported that the oxygen saturation of larger retinal arteries was greater in eyes with retinal vein occlusion (RVO) than in unaffected eyes [20, 21]. Furthermore, a modest, although not statistically significant, decrease in arterial oxygen saturation, measured using traditional retinal oximetry methods, was observed after anti-VEGF therapy in eyes with macular edema associated with RVO [22]. A second possible explanation is that VEGF plays an important role in the remodeling of the paCFZ. Venous occlusion was reported to cause retinal ischemia and upregulate proangiogenic factors in the retina, such as VEGF, which induces the adhesion and aggregation of leukocytes [23]. The consequent exacerbated retinal endothelial damage may result in the closure of capillaries near the retinal arteries and expansion of the paCFZ. Previous studies have suggested that blocking VEGF might lead to reperfusion of nonperfused areas and improve retinal blood flow in eyes with RVO [24, 25]. Therefore, we hypothesize that anti-VEGF therapy further favors capillary reperfusion along the major arteries and hence reduces the paCFZ in eyes with macular edema secondary to BRVO.

We found negligible changes in the areas of the major retinal arteries following anti-VEGF therapy. These findings are consistent with those reported by Im et al., who observed unremarkable changes in the artery diameters during a long-term follow-up after anti-VEGF therapy [26]. It is supposed that the thick artery walls showed increased resistance to the vasoconstrictive effect of anti-VEGF therapy [26, 27]. Accordingly, we calculated the P/A ratios in our study to reduce the possible influence of the variability in the branches of retinal arteries. It turned out that the P/A ratios were significantly larger in the eyes with ischemic BRVO than nonischemic BRVO before and after anti-VEGF treatment, indicating that the P/A ratio may be a sensitive biomarker for distinguishing the different types of BRVO. Furthermore, the P/A ratios, but not the paCFZ areas and artery areas, were significantly correlated with the baseline CRT and its improvement over time. Few studies have evaluated the relationship between the P/A ratios and CRT [13]. Severe macular edema and increased CRT may suggest greater impairment of the blood-retinal barrier with higher concentrations of vasopermeability mediators like VEGF [28, 29]. As mentioned above, VEGF levels may play a key role in the changes of paCFZ parameters. Anti-VEGF therapy and the resultant decrease in intraocular VEGF levels could occur in parallel with the changes in the P/A ratios and CRT. Therefore, larger baseline P/A ratios may predict greater decreases in CRT after anti-VEGF therapy. This may also explain, at least in part, why the P/A ratios were significantly correlated with the number of ranibizumab injections.

We previously found that smaller P/A ratios in the first- and second-order retinal arteries at baseline were significantly associated with better BCVA at baseline [13]. Visual acuity may be preserved in patients with mild occlusion of a branch vein with less-prominent retinal ischemia and a small P/A ratio. Here, we found that a smaller P/A ratio was associated with a greater improvement in BCVA at 3, 6, and 12 months upon univariate analyses, and with a better BCVA at 12 months after therapy after the multivariate analysis. However, there was no significant correlation between baseline FAZ and BCVA at 12 months. A few studies have previously reported a correlation between visual acuity and FAZ, but the conclusions have been inconsistent [8, 30–32]. Some studies have found that enlargement of the FAZ was associated with reduced visual acuity [8, 30]. Wakabayashi et al. reported that a smaller FAZ area was associated with better visual acuity after treatment of macular edema in BRVO, but it was not a significant variable in the multivariate linear regression model [31]. Song et al. found that there was no significant correlation between FAZ and visual acuity in BRVO before and after anti-VEGF therapy [32], which was consistent with the results of our studies. These discrepancies may be related to the interindividual variability in the FAZ size [15], various measurement methods [30, 31], OCTA instrument [32], and different stages of BRVO in which the acute macular edema with masked or displaced macular vessels may affect the accurate measurement of FAZ [31]. In this study, the paCFZ parameters were measured along the major retinal arteries and less affected by the macular edema or hemorrhage, which might be an advantage of the paCFZ biomarker. Consistent with previous reports [33, 34], our results showed that baseline BCVA was independently associated with the final BCVA after anti-VEGF therapy. Although duration of symptoms and baseline CRT were associated with the final BCVA in univariate analyses, they were not significant variables in the multivariate linear regression model. We included treatment-naïve patients with a symptom duration of less than 3 months, which is shorter than those in the BRAVO and VIBRANT studies (≤ 12 months) [35, 36]. We also note that the treatment regimens varied in the BRAVO and VIBRANT studies [35, 36], and a 3 + PRN regimen was used in our study. Therefore, these differences may help explain the conflicting results of the prognostic factor analysis in this study.

This retrospective study has some limitations. First, the SS-OCTA parameters were measured without corrections for the possible image magnification errors due to insufficient axial length data. Second, subjective factors may have influenced manual tracing of the paCFZ and retinal arteries to measure their areas, although the intra-observer and inter-observer agreements were high. Finally, we did not assess whether macular OCT features other than CRT were associated with BCVA and paCFZ parameters.

## Conclusions

The current study using wide-field SS-OCTA images suggests that anti-VEGF therapy can lead to a significant improvement in the paCFZ parameters in BRVO and patients with smaller baseline P/A ratios on SS-OCTA tend to have better final BCVA at 12 months.

## Supplementary Information

**Additional file 1: Table S1.** Intra-observer repeatability and inter-observer reproducibility of paCFZ and artery area measurement. **Table S2.** Changes of the paCFZ areas, artery areas and P/A ratios in healthy contralateral eyes during the follow-up period. **Table S3.** Simple regression analysis of baseline paCFZ area and artery area with central retinal thickness and best-corrected visual acuity.

## Data Availability

All data generated or analyzed during this study are included in this published article and its Additional files.

## Abbreviations

SS-OCTA: Swept-source optical coherence tomography angiography
VEGF: Vascular endothelial growth factor
BRVO: Branch retinal vein occlusion
P/A: PaCFZ area to artery area
BCVA: Best-corrected visual acuity
IOP: Intraocular pressure
ETDRS: Early Treatment Diabetic Retinopathy Study
FA: Fluorescein angiography
paCFZ: Periarterial capillary-free zone
FAZ: Foveal avascular zone
IVR: Intravitreal ranibizumab
SD-OCT: Spectral-domain optical coherence tomography
CRT: Central retinal thickness
PRN: Pro re nata
ICC: Intraclass correlation coefficients
ANOVA: Analysis of variance

## References

[1] Rogers S, McIntosh RL, Cheung N, Lim L, Wang JJ, Mitchell P (2010). The prevalence of retinal vein occlusion: pooled data from population studies from the United States, Europe, Asia, and Australia. Ophthalmology.

[2] Noma H, Funatsu H, Yamasaki M, Tsukamoto H, Mimura T, Sone T (2005). Pathogenesis of macular edema with branch retinal vein occlusion and intraocular levels of vascular endothelial growth factor and interleukin-6. Am J Ophthalmol.

[3] Campochiaro PA, Heier JS, Feiner L, Gray S, Saroj N, Rundle AC (2010). Ranibizumab for macular edema following branch retinal vein occlusion: six-month primary end point results of a phase III study. Ophthalmology.

[4] Shalchi Z, Mahroo O, Bunce C, Mitry D (2020). Anti-vascular endothelial growth factor for macular oedema secondary to branch retinal vein occlusion. Cochrane Database Syst Rev.

[5] Ang JL, Ah-Moye S, Kim LN, Nguyen V, Hunt A, Barthelmes D (2020). A systematic review of real-world evidence of the management of macular oedema secondary to branch retinal vein occlusion. Eye.

[6] Yamada R, Nishida A, Shimozono M, Kameda T, Miyamoto N, Mandai M (2015). Predictive factors for recurrence of macular edema after successful intravitreal bevacizumab therapy in branch retinal vein occlusion. Jpn J Ophthalmol.

[7] Spaide RF, Fujimoto JG, Waheed NK, Sadda SR, Staurenghi G (2018). Optical coherence tomography angiography. Prog Retin Eye Res.

[8] Samara WA, Shahlaee A, Sridhar J, Khan MA, Ho AC, Hsu J (2016). Quantitative optical coherence tomography angiography features and visual function in eyes with branch retinal vein occlusion. Am J Ophthalmol.

[9] Freund KB, Sarraf D, Leong BCS, Garrity ST, Vupparaboina KK, Dansingani KK (2018). Association of optical coherence tomography angiography of collaterals in retinal vein occlusion with major venous outflow through the deep vascular complex. JAMA Ophthalmol.

[10] His W (1880). Abbildungen uber des Gefaszsystem der menschlichen Netzhaut und derjenigen des Kaninchens. Arch f Anat u Entwickelungsg.

[11] Michaelson I, Campbell A (1940). The anatomy of the finer retinal vessels, and some observations on their significance in certain retinal diseases. Trans Ophthalmol Soc UK.

[12] Claxton S, Fruttiger M (2003). Role of arteries in oxygen induced vaso-obliteration. Exp Eye Res.

[13] Tang W, Guo J, Zhuang X, Zhang T, Wang L, Wang K (2021). Wide-field swept-source optical coherence tomography angiography analysis of the periarterial capillary-free zone in branch retinal vein occlusion. Transl Vis Sci Technol.

[14] Li H, Ding X, Lu L, Yang J, Ma J (2019). Morphometry of the normal retinal periarteral capillary-free zone and changes during severe nonproliferative diabetic retinopathy. Clin Hemorheol Microcirc.

[15] Arthur E, Elsner AE, Sapoznik KA, Papay JA, Muller MS, Burns SA (2019). Distances from capillaries to arterioles or venules measured using OCTA and AOSLO. Invest Ophthalmol Vis Sci.

[16] The Branch Vein Occlusion Study Group (1984). Argon laser photocoagulation for macular edema in branch vein occlusion. Am J Ophthalmol.

[17] Yeung L, Wu WC, Chuang LH, Wang NK, Lai CC (2019). Novel optical coherence tomography angiography biomarker in branch retinal vein occlusion macular edema. Retina.

[18] Ishii H, Shoji T, Yoshikawa Y, Kanno J, Ibuki H, Shinoda K (2019). Automated measurement of the foveal avascular zone in swept-source optical coherence tomography angiography images. Transl Vis Sci Technol.

[19] Ishibazawa A, Mehta N, Sorour O, Braun P, Martin S, Alibha AY (2019). Accuracy and reliability in differentiating retinal arteries and veins using widefield en face OCT angiography. Transl Vis Sci Technol.

[20] Jeppesen SK, Bek T (2017). Retinal oxygen saturation correlates with visual acuity but does not predict outcome after anti-VEGF treatment in central retinal vein occlusion. Invest Ophthalmol Vis Sci.

[21] Yang JY, You B, Wang Q, Chan SY, Jonas JB, Wei WB (2017). Retinal vessel oxygen saturation in healthy subjects and early branch retinal vein occlusion. Int J Ophthalmol.

[22] Feng XX, Li C, Shao WW, Yuan YG, Qian XB, Zheng QS (2018). Intravitreal anti-VEGF agents, oral glucocorticoids, and laser photocoagulation combination therapy for macular edema secondary to retinal vein occlusion: preliminary report. Int J Ophthalmol.

[23] Kim I, Moon SO, Kim SH, Kim HJ, Koh YS, Koh GY (2001). Vascular endothelial growth factor expression of intercellular adhesion molecule 1 (ICAM-1), vascular cell adhesion molecule 1 (VCAM-1), and E-selectin through nuclear factor-kappa B activation in endothelial cells. J Biol Chem.

[24] Suzuki N, Hirano Y, Tomiyasu T, Esaki Y, Uemura A, Yasukawa T (2016). Retinal hemodynamics seen on optical coherence tomography angiography before and after treatment of retinal vein occlusion. Invest Ophthalmol Vis Sci.

[25] Mir TA, Kherani S, Hafiz G, Scott AW, Zimmer-Galler I, Wenick AS (2016). Changes in retinal nonperfusion associated with suppression of vascular endothelial growth factor in retinal vein occlusion. Ophthalmology.

[26] Im JC, Shin JP, Kim IT, Park DH (2016). Recurrence of macular edema in eyes with branch retinal vein occlusion changes the diameter of unaffected retinal vessels. Graefes Arch Clin Exp Ophthalmol.

[27] Michelson G, Warntges S, Baleanu D, Welzenbach J, Ohno-Jinno A, Pogorelov P (2007). Morphometric age-related evaluation of small retinal vessels by scanning laser Doppler flowmetry: determination of a vessel wall index. Retina.

[28] Miyamoto K, Khosrof S, Bursell SE, Moromizato Y, Aiello LP, Ogura Y (2000). Vascular endothelial growth factor (VEGF)-induced retinal vascular permeability is mediated by intercellular adhesion molecule-1 (ICAM-1). Am J Pathol.

[29] Noma H, Minamoto A, Funatsu H, Tsukamoto H, Nakano K, Yamashita H (2006). Intravitreal levels of vascular endothelial growth factor and interleukin-6 are correlated with macular edema in branch retinal vein occlusion. Graefes Arch Clin Exp Ophthalmol.

[30] Parodi M, Visintin F, Della Rupe P, Ravalico G (1995). Foveal avascular zone in macular branch retinal vein occlusion. Int Ophthalmol.

[31] Wakabayashi T, Sato T, Hara-Ueno C, Fukushima Y, Sayanagi K, Shiraki N (2017). Retinal microvasculature and visual acuity in eyes with branch retinal vein occlusion: imaging analysis by optical coherence tomography angiography. Invest Ophthalmol Vis Sci.

[32] Song S, Yu X, Zhang P, Dai H (2021). Changes in macular microvascular structure in macular edema secondary to branch retinal vein occlusion treated with anti-vascular endothelial growth factor for one year. J Ophthalmol.

[33] Winegarner A, Wakabayashi T, Fukushima Y, Sato T, Hara-Ueno C, Busch C (2018). Changes in retinal microvasculature and visual acuity after antivascular endothelial growth factor therapy in retinal vein occlusion. Invest Ophthalmol Vis Sci.

[34] Inagaki M, Hirano Y, Yasuda Y, Kawamura M, Suzuki N, Yasukawa T (2021). Twenty-four month results of intravitreal ranibizumab for macular edema after branch retinal vein occlusion: visual outcomes and resolution of macular edema. Semin Ophthalmol.

[35] Brown DM, Campochiaro PA, Bhisitkul RB, Ho AC, Gray S, Saroj N (2011). Sustained benefits from ranibizumab for macular edema following branch retinal vein occlusion: 12-month outcomes of a phase III study. Ophthalmology.

[36] Clark WL, Boyer DS, Heier JS, Brown DM, Haller JA, Vitti R (2016). Intravitreal aflibercept for macular edema following branch retinal vein occlusion: 52-week results of the VIBRANT study. Ophthalmology.

