# Supplementary material for: Wide-field swept-source OCT angiography of the periarterial capillary-free zone before and after anti-VEGF therapy for branch retinal vein occlusion
Source: Eye Vis (Lond). 2022 Jul 2;9:25. doi: 10.1186/s40662-022-00297-z (PMC9250258; doi:10.1186/s40662-022-00297-z)
Supplement: Supplementary file 1 — Additional file 1: Table S1. Intra-observer repeatability and inter-observer reproducibility of paCFZ and artery area measurement. Table S2. Changes of the paCFZ areas, artery areas and P/A ratios in healthy contralateral eyes during the follow-up period. Table S3. Simple regression analysis of baseline paCFZ area and artery area with central retinal thickness and best-corrected visual acuity. [file 40662_2022_297_MOESM1_ESM.docx]

**Table S1** Intra-observer repeatability and inter-observer reproducibility of paCFZ and artery area measurement

| Variables | ICC (95% CI) for intra-observer repeatability | | ICC (95% CI) for inter-observer reproducibility | |
| --- | --- | --- | --- | --- |
|  | BRVO eyes | Fellow eyes | BRVO eyes | Fellow eyes |
| First-order |  |  |  |  |
| Artery area | 0.970 (0.948–0.982) | 0.938 (0.896–0.964) | 0.959 (0.931–0.976) | 0.925 (0.875–0.956) |
| PaCFZ | 0.985 (0.974–0.991) | 0.960 (0.932–0.977) | 0.968 (0.946–0.981) | 0.947 (0.911–0.969) |
| Second-order |  |  |  |  |
| Artery area | 0.937 (0.894–0.963) | 0.939 (0.897–0.964) | 0.945 (0.907–0.968) | 0.939 (0.897–0.964) |
| PaCFZ | 0.934 (0.889–0.961) | 0.965 (0.941–0.980) | 0.961 (0.933–0.977) | 0.957 (0.927–0.975) |

*paCFZ =* periarterial capillary-free zone; *ICC =* intraclass correlation coefficient; *CI =* confidence interval; *BRVO =* branch retinal vein occlusion

**Table S2** Changes of the paCFZ areas, artery areas and P/A ratios in healthy contralateral eyes during the follow-up period

| Variables | BL | 12m | *P** |
| --- | --- | --- | --- |
| First-order artery |  |  |  |
| PaCFZ area (mm^2^) | 0.45 ± 0.15 | 0.45 ± 0.16 | 0.23 |
| Artery area (mm^2^) | 0.53 ± 0.14 | 0.54 ± 0.15 | 0.06 |
| P/A ratio | 0.84 ± 0.22 | 0.83 ± 0.22 | 0.06 |
| Second-order artery |  |  |  |
| PaCFZ area (mm^2^) | 0.75 ± 0.25 | 0.76 ± 0.25 | 0.21 |
| Artery area (mm^2^) | 0.65 ± 0.23 | 0.65 ± 0.22 | 0.55 |
| P/A ratio | 1.25 ± 0.53 | 1.26 ± 0.53 | 0.22 |

*BL =* baseline; *paCFZ =* periarterial capillary-free zone; *P/A =* paCFZ area to artery area.

* Paired t-test

**Table S3** Simple regression analysis of baseline paCFZ area and artery area with CRT and BCVA

| Variables | paCFZ area | | | | Artery area | | | |
| --- | --- | --- | --- | --- | --- | --- | --- | --- |
|  | First-order | | Second-order | | First-order | | Second-order | |
|  | r | *P* | r | *P* | r | *P* | r | *P* |
| Baseline CRT | 0.254 | 0.064 | 0.066 | 0.637 | 0.077 | 0.582 | −0.191 | 0.166 |
| CRT improvement |  |  |  |  |  |  |  |  |
| At 3 months | 0.207 | 0.134 | 0.079 | 0.572 | 0.055 | 0.693 | −0.139 | 0.317 |
| At 6 months | 0.132 | 0.343 | 0.102 | 0.461 | −0.008 | 0.951 | −0.119 | 0.392 |
| At 12 months | 0.164 | 0.237 | 0.068 | 0.627 | −0.007 | 0.960 | −0.179 | 0.196 |
| Baseline BCVA | 0.038 | 0.787 | 0.046 | 0.739 | 0.218 | 0.113 | 0.262 | 0.056 |
| BCVA improvement | |  |  |  |  |  |  |  |
| At 3 months | −0.097 | 0.487 | 0.056 | 0.690 | 0.268 | 0.050 | 0.259 | 0.059 |
| At 6 months | −0.007 | 0.958 | 0.080 | 0.564 | 0.149 | 0.281 | 0.189 | 0.172 |
| At 12 months | 0.958 | 0.157 | 0.078 | 0.576 | 0.056 | 0.690 | 0.255 | 0.063 |

*P/A =* paCFZ area to artery area; *paCFZ =* periarterial capillary-free zone; *CRT =* central retinal thickness; *BCVA =* best-corrected visual acuity; *ETDRS =* Early Treatment Diabetic Retinopathy Study
